# Measuring tooth size discrepancies using Bolton analysis: a comparative cross-sectional study among major ethnicity in Malaysia

**DOI:** 10.1186/s12903-023-03185-7

**Published:** 2023-08-02

**Authors:** Aida Nur Ashikin Abd Rahman, Siti Adibah Othman, Anand Marya

**Affiliations:** 1 Centre for Paediatric Dentistry and Orthodontic Studies, Faculty of Dentistry, Universiti Teknologi MARA, Selangor Branch, Sungai Buloh Campus, Jalan Hospital, Sungai Buloh, Selangor, 47000 Malaysia; 2 Department of Paediatric Dentistry and Orthodontics, Faculty of Dentistry, Universiti Malaya, Kuala Lumpur, 50603 Malaysia; 3 Department of Orthodontics, Faculty of Dentistry, University of Puthisastra, No. 55, St. 180, Phnom Penh, 12211 Cambodia

**Keywords:** Tooth size discrepancy, Bolton analysis, Anterior ratio, Overall ratio

## Abstract

**Background:**

The Bolton analysis is one of the commonly used tooth size analysis or diagnostic tools in deriving a treatment plan for orthodontic patients. Many studies have indicated and concluded that normal measurements for one group should not be considered normal for other ethnic groups. The aims and objectives of this study were to investigate the applicability of Bolton’s ratios in the orthodontic population of Malaysian main ethnics, Malay, Chinese, and Indians. Comparisons were made in terms of size and distribution of tooth size discrepancy in the Malaysian orthodontic population and the findings were converted in terms of millimeters.

**Methods:**

Hundred fifty pre-orthodontic study casts comprised of 52 Malay, 54 Chinese, and 44 Indian patients were selected. Digital calipers (Fowler Pro-Max) linked to Hamilton Tooth Arch Software were used to measure the tooth width and ratios. Statistical analysis was carried out to test for gender differences (independent *t*-test), to identify the effects of malocclusion and ethnic groups (Two-way ANOVA), and to compare the means of the current study with Bolton’s standards (one sample t-test).

**Results:**

This study showed that there was no significant difference between the genders of the sample of each ethnicity. There was no correlation found between ethnic groups and malocclusion classes. There was a significant difference when comparing Bolton values with the Malay sample for both ratios. It was found that more Malay subjects presented with maxillary excess contrary to Chinese and Indians who presented more maxillary deficiency for the anterior and overall ratio.

**Conclusion:**

There was a significant difference found between the TSD of the three major ethnicities in Malaysia. The Bolton standards can be applied to Malaysian Chinese and Indians but not to Malays orthodontic populations for both anterior and overall ratios. Subsequently, a specific standard should be used for the Malays orthodontic population. It was found that more Malay subjects presented with maxillary excess contrary to Chinese and Indians who presented more maxillary deficiency for the anterior and overall ratio.

## Background

The Bolton analysis is one of the commonly used tooth size analysis or diagnostic tools in deriving a treatment plan for orthodontic patient. However, Bolton [1] had carried out the study in 55 excellent occlusion cases among Caucasians. From his study, he formulated that overall ratio of 91.3 (± 1.91%) and anterior ratio of 77.2 (± 1.65%) will achieve a good interdigitation with good occlusion.

Abd-Rahman and Othman [2] stated that numerous studies have indicated that normal measurements for one ethnic group should not be considered normal for other ethnic groups. A possible indication for population-specific standards are necessary for clinical assessments of each population, namely the Malaysian population.

Studies carried out to Asian population which also demonstrated differences among malocclusion groups among southern Chinese children in Hong Kong [3] and Japanese orthodontic population [4, 5] have suggested a different standard than Bolton’s although a study done among Class I Singaporean Chinese found a comparable result with Bolton’s standard [6].

Previous studies have shown that tooth size ratios are ethnicity, and sex-specific and that populations differ with respect to interarch tooth-size relationships, and differences in tooth sizes [5, 7–12]. Tooth size exhibits a continuous range of variation among individuals and between populations. The variation in tooth size is influenced by genetic, environmental factors, ethnicity and gender. Several studies have demonstrated that mesiodistal crown diameters of males are larger than females [7] but however, this does not mean that they have larger tooth size ratios or an increased prevalence of tooth size discrepancies (TSD) [12].

The high prevalence of TSD in orthodontic population in relative to the general population has been well documented [12, 13]. Findings of the norms will be a guide in determining the TSD in orthodontic populations that require orthodontic treatment. These values will be in turn converted in millimeters for decision in the treatment planning whether interproximal approximation is required for arch coordination and ideal interdigitation or reduction in tooth structures or even extractions as an option if severe TSD are present.

The second part of Bolton’s study [14] described the clinical application of a tooth size analysis. Clinical application was discussed in depth in term of calculation of the arch length comparisons and determination of TSD in millimeters. The overall ratio was calculated from the summed of the greatest mesiodistal measurements of the teeth in each arch from first molar to first molar. The anterior ratio was calculated from the summed of the greatest mesiodistal measurements of the six anterior teeth in each arch. The tooth size measurement on the dental plaster cast by a caliper was reported to give an additional advantage of repeating the measurement with good consistency and accuracy. Conventional vernier caliper was found to be comparably accurate as digital measurement [15].

### Aims of the study

A) To compare the mean of anterior and overall ratios among the three majority ethnic groups in Malaysia; Malay, Chinese and Indian.
B) To compare the mean of anterior and overall ratios of each ethnicity with Bolton’s original study.
C) To investigate the size and distribution of TSD in the orthodontic population and convert the findings in terms of millimeters.

## Methods

### Sample selection

Determined sample size of pretreatment study casts from each ethnic of who have sought orthodontic treatment in orthodontic treatment in Dental Faculty, in Kuala Lumpur were selected from one cohort (cross sectional for the number of patients seeking orthodontic treatment between January to June. The sample size for the first objective was calculated using G*Power following the rules for F test, using ANOVA: fixed effects, special, main effects and interactions. Alpha was set at 0.08, power at 80%, effect size = 0.5 (medium), numerator df = 2 and number of groups = 6, which requires a total sample of 42 subjects. The sample size for the second objective was calculated using *t*-tests of means difference from constant (one sample case) with alpha set at 0.05, power at 90% and effect size = 0.5 giving total sample size of 36 samples for each group. In this study, we have included 150 patients with 300 study casts that fit the inclusion were obtained to satisfy the sample size calculation with 54 Chinese, 52 Malays and 44 Indians.

### Selection criteria

Inclusion criteria;

1. Malay, Chinese, and Indian ethnicity. Ethnicity determinations were according to their name and race in the registration form and interview conducted to patients and parents.
2. Fully erupted and complete permanent dentitions from first molar to contralateral first molar.
3. Good quality pretreatment study casts.

Exclusion criteria;

1. Clinically visible dental caries, proximal restorations (Class II amalgam or composite), build ups, crowns and onlays that affect the tooth’s mesiodistal diameter.
2. Congenital defects or deformed teeth.
3. Obvious interproximal or occlusal wear of teeth.
4. Previous or ongoing orthodontic treatment.
5. Patient who came from mixed race and intermarriage.

### Data collection

**Fig. 1:**
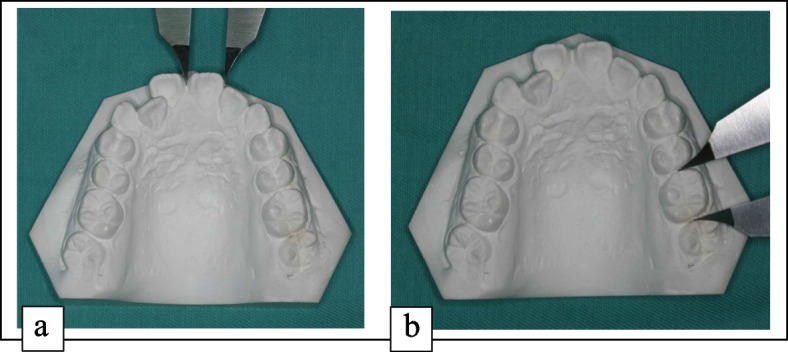
Measuring the **a** Anterior and **b** Overall Ratio

Measurements of the greatest mesiodistal width of each tooth were taken with the caliper’s tips held perpendicular to the long axis of each tooth from the first molar to the first molar of each cast. The mesiodistal widths from the contact points of canine to canine for anterior ratio and first molar to first molar for overall ratio **(**Fig. 1**)** were measured on each cast to the nearest 0.01 mm, by using digital calipers (Fowler Pro-Max Calipers) linked to Hamilton Arch Tooth System software (HATS). The HATS software will calculate the anterior and overall ratio of each pair of orthodontic study cast using Bolton analysis. The tooth size corrections will also be calculated by the HATS software. The readings were recorded in the data collection sheet for further statistical management.

### Applications of bolton analysis

The value of correction in millimetres can be taken from the data collection sheet or calculated. Total corrections of anterior and overall ratios were calculated using a given formula and converted in term of millimetres. Bolton ratios were calculated in term of millimetres by employing the Bolton’s anterior and overall ratios’ formula and subtracting the solution from the existing summations of mesiodistal width of upper or lower teeth. The formula then further simplified and applied for accurate calculations in the SPSS as shown below;

**Anterior ratio:**

$$\frac{\mathbf X\;(\mathbf{ideal}\boldsymbol\;\mathbf{mandibular}\boldsymbol\;\boldsymbol''\mathbf6\boldsymbol'')}{(\mathbf{maxillary}\boldsymbol\;\boldsymbol'\boldsymbol'\mathbf6\boldsymbol'\boldsymbol')}\times\mathbf{100}=\mathbf{77}\boldsymbol.\mathbf2\;\boldsymbol(\mathbf{Bolton}\boldsymbol'\mathbf s\boldsymbol\;\mathbf{standard})$$

$$\frac{(\mathbf{mandibular}\boldsymbol\;\boldsymbol'\boldsymbol'\mathbf6\boldsymbol'\boldsymbol')}{\mathbf Y\;(\mathbf{ideal}\boldsymbol\;\mathbf{maxillary}\boldsymbol\;\boldsymbol'\boldsymbol'\mathbf6\boldsymbol'\boldsymbol')}\times\mathbf{100}\boldsymbol=\mathbf{77}\boldsymbol.\mathbf2\;(\mathbf{Bolton}\boldsymbol'\mathbf s\boldsymbol\;\mathbf{standard})$$

$$Upper\;correction\;(mm)=(Sum\;of\;maxillary\;''6''\;teeth)-\lbrack(Sum\;of\;mandibular\;''6''\;teeth)\;\div\;0.772\rbrack$$

$$Lower\;correction\;(mm)\;=\;(Sum\;of\;mandibular\;''6''\;teeth)\;-\;\lbrack(sum\;of\;maxillary\;''6''\;teeth)\;\times\;0.772\rbrack$$

**Overall ratio:**

$$\frac{\mathbf X\boldsymbol\;(\mathbf{ideal}\boldsymbol\;\mathbf{mandibular}\boldsymbol\;\boldsymbol'\boldsymbol'\mathbf{12}\boldsymbol'\boldsymbol')}{(\mathbf{maxillary}\boldsymbol\;\boldsymbol'\boldsymbol'\mathbf{12}\boldsymbol'\boldsymbol')}\times\boldsymbol\;\mathbf{100}\boldsymbol\;\boldsymbol=\boldsymbol\;\mathbf{91}\boldsymbol.\mathbf3\;(\mathbf{Bolton}\boldsymbol'\mathbf s\boldsymbol\;\mathbf{standard})$$

$$\frac{(\mathbf{mandibular}\boldsymbol\;\boldsymbol'\boldsymbol'\mathbf{12}\boldsymbol'\boldsymbol')}{\mathbf Y\;(\mathbf{ideal}\boldsymbol\;\mathbf{maxillary}\boldsymbol\;\boldsymbol'\boldsymbol'\mathbf{12}\boldsymbol'\boldsymbol')}\times\mathbf{100}\boldsymbol\;\boldsymbol=\boldsymbol\;\mathbf{91}\boldsymbol.\mathbf3\boldsymbol\;\boldsymbol(\mathbf{Bolton}\boldsymbol'\mathbf s\boldsymbol\;\mathbf{standard}\boldsymbol)$$

$$Upper\;correction\;(mm)\;=\;(Sum\;of\;maxillary\;''12''\;teeth)\;-\;\lbrack(Sum\;of\;mandibular\;''12''\;teeth)\;\div\;0.913\rbrack$$

$$Lower\;correction\;(mm)\;=\;(Sum\;of\;mandibular\;''12''\;teeth)\;-\;\lbrack(sum\;of\;maxillary\;''12''\;teeth)\;\times\;0.913\rbrack$$

### Reliability and reproducibility of the measurements

Two pairs of dental casts selected above were measured by one inter-examiner for data calibration as a gold standard using reliability analysis and intra class correlation by expertise in the field for measurement calibration. A high correlation of 0.994 between the principle investigator and the reference investigator was achieved. For intra-examiner reliability analysis, ten pairs of dental casts were randomly selected systematically by randomization using Statistical Package for the Social Sciences (SPSS) software. Each cast was measured at two separate occasions in two weeks’ interval by the principal investigator. The paired-sample *t*-test was used to evaluate the systematic error, and there was no statistically significant systematic error noted with *p* > 0.05. Our finding shows a very high correlation of Cronbach’s Alpha 0.995 between the first and second measurements and indicates that examiner was highly consistent in the measurement of the parameter.

### Statistical analysis

Skewness test shows data to be within 0 ± 2 which is within normal data distribution range. Kolmogorov–Smirnov test of normality was also carried out for every ethnic group and showed no statistic significant (*p* > 0.05), hence data normality was assumed and further parametric test were carried out. Independent *t-*test was carried out to test for gender differences, two-way ANOVA to identify the effects of malocclusion and ethnic groups and one-sample *t*-test to compare the means of current study with Bolton’s standards.

The data collected from the records were evaluated to determine the percentage of patients who had TSDs which were within one, within two, or greater than two standard deviations from Bolton's mean. A mean, median, range, standard deviation, standard error of the mean, and coefficient of variation were calculated for both the overall "12" ratio and anterior *"6"* ratio. Two-way analyses of variance (ANOVA) were used to correlate the interactions between ethnic and anterior ratio and overall ratio.

### Applications of bolton analysis

The value of correction in millimeters was calculated. Total corrections of anterior and overall ratios were calculated using a given formula and converted in term of millimeters. Bolton ratios were calculated in term of millimeters by employing the Bolton’s anterior and overall ratios’ formula and subtracting the solution from the existing summations of mesiodistal width of upper or lower teeth.

## Results

From Table 1 it was found that there was no gender difference (*p* > 0.05) within combination samples, and within all ethnicity for female and male and for anterior and overall ratios. Further statistical analyses were carried out without splitting the gender. A consistent trend of relatively higher mean was observed in male subject compared to female subjects in both anterior and overall ratio among all ethnic groups. However, these differences were not of statistically significant.

**Table 1:** Independant *t-*test between gender among different ethnic group in Malaysia

Variables	Mean (SD)	Mean (SD)	Mean Difference (*df*)	*P* value
**Malay (*n* = 52)**	**Male (*n* = 13)**	**Female (*n* = 39)**		
**Anterior Ratio**	78.45 (1.93)	78.29 (2.30)	0.16	0.821
**Overall Ratio**	92.73 (1.22)	91.99 (1.63)	0.74	0.138
**Chinese (*n* = 54)**	**Male (*n* = 15)**	**Female (*n* = 39)**		
**Anterior Ratio**	76.84 (1.93)	76.42 (2.66)	0.42	0.577
**Overall Ratio**	91.38 (1.37)	90.64 (2.16)	0.74	0.142
**Indian (*n* = 44)**	**Male (*n* = 19)**	**Female (*n* = 25)**		
**Anterior Ratio**	78.25 (3.17)	77.21 (2.54)	1.04	0.237
**Overall Ratio**	91.26 (2.16)	91.18 (1.75)	0.08	0.891

From Table 2, there were statistical differences found between ethnicity but no interaction found between ethnicity and malocclusion types in the anterior (*p* = 0.186) or overall ratios (*p* = 0.073) from two-way ANOVA analyses. Statistical difference was found between ethnicity and post-hoc test was carried out. From the Bonferroni post-hoc test, shows that there are significant differences found between Malays and Chinese (*P* < 0.001) and between Malays and Indian subjects in the overall ratio (*P* = 0.029).

**Table 2:** Two-way ANOVA Post hoc test of ethnic group for overall ratio

Overall Ratio						
**Variables**	**Std Error (SE)**			***p* value**	**95% Confidence Interval**	
					**Lower bound**	**Upper bound**
**Malay**						
**Chinese**		0.35		< 0.001*	0.489	2.164
**Indian**		0.36		0.029*	0.074	1.841
**Chinese**						
**Malay**		0.35	< 0.001*		-2.164	-0.488
**Indian**		0.36	0.928		-1.245	0.507
**Indian**						
**Malay**		0.36	0.029*		-1.841	-0.074
**Chinese**		0.36	0.928		-0.507	1.245

^*^statistical significant *p* < 0.05

**Fig. 2:**
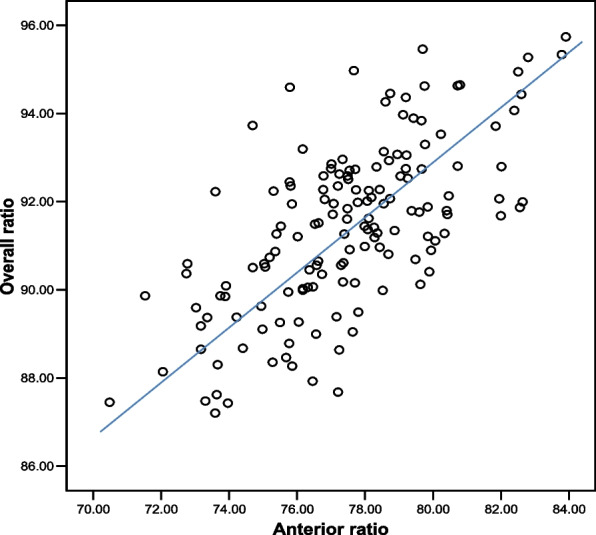
Scatterplot to visualize the distribution of the correlation data

Table 3 shows one sample *t*-test was used to test the applicability of Bolton’s value to different ethnics in Malaysia samples. It was found that there was no significant difference found between all the groups with Bolton standard except Malay. Figure 2 shows the scatterplot to visualize the distribution of the correlation data which is shown to have a linear relationship between the two variables. Using Pearson’s correlation analysis, it was found that the correlation between anterior and total tooth width ratios was moderate (Pearson’s correlation 0.665, *P* < 0.001).

**Table 3:** Comparison of anterior and overall ratios between Malaysian majority ethnics and Bolton standards

	**Anterior Ratio**			**Overall Ratio**		
**Ethnic**	**Malay**	**Chinese**	**Indian**	**Malay**	**Chinese**	**Indian**
**n**	52	54	44	52	54	44
**Mean (SD)**	78.33 (2.19)	76.53 (2.47)	77.66 (2.84)	92.18 (1.56)	90.85 (1.99)	91.22 (1.91)
**Mean Range**	76.14–80.52	74.06–79.00	74.82–80.50	90.62–93.74	88.86–92.84	89.31–93.13
**Bolton (SD)**	77.20 (1.65)			91.30 (1.91)		
***P*** **value**	< 0.001*	0.052	0.289	< 0.001*	0.101	0.777

^*^Statistically significant

In this table, comparing Malay’s samples and Bolton’s standard, showed highly significant differences between the Malay anterior ratio (*p* < 0.001) and overall ratio (*p* < 0.001) which suggest non applicability of Bolton’s value to Malay ethnic.

However, Bolton ratios are found not to have any statistically significant differences when compared with the Chinese and Indians anterior and overall ratios as shown in Table 3 with* p* = 0.052 and *p* = 0.289 for anterior ratio and *p* = 0.101 and *p* = 0.777 for overall ratio in Chinese and Indian subjects respectively.

Mean range of Malay subjects are found to be on the higher limit and beyond of Bolton’s range of both anterior and overall ratios. For anterior ratio, mean range for Malay ethnic group, are between 76.14 to 80.52 compared to Bolton’s value between 75.55 to 78.85. For overall ratio of Malay ethnic group, the mean ranging from between 90.62 to 93.74 compared to Bolton’s value between 89.39 to 93.21 **(**Table 3**)**.

For overall ratio **(**Table 4**),** More Malay subjects present with maxillary excess (15.4%) and least percentage of subjects present with either mandibular excess in this group (1.9%). Contrary to findings in Malay ethnic group, more subjects presented with maxillary deficiency with 20.4% in Chinese and 11.4% in Indian respectively. However, least percentage of subjects presented with maxillary excess in Chinese (3%) as compared to Indian which have the least mandibular excess problem (4.5%) similarly as found in Malay.

**Table 4:** Percentage of subjects with clinically significant tooth-width discrepancies larger than 2.0 mm according to ethnic group

	***Low-range proportion***		***High range proportion***	
	Mandibular deficiency	Maxillary excess	Mandibular excess	Maxillary deficiency
**Anterior ratio**				
***Malay***	11.5%	15.4%	1.9%	3.8%
***Chinese***	3.7%	3%	5.6%	20.4%
***Indian***	9.1%	9.1%	4.5%	11.4%
***Overall ratio***				
***Malay***	19.2%	19.2%	1.9%	5.8%
***Chinese***	11.1%	13%	24.1%	27.8%
***Indian***	11.4%	11.4%	9.1%	13.6%

Tables 5 and 6 illustrate the percentage of upper and lower correction in millimetres for both anterior and overall ratios of each ethnic group. There were mixed combinations of upper and lower corrections required to match the Bolton ratios.

**Table 5:** Percentage of subjects according to ethnicity requiring upper and lower anterior correction

*Correction*	Upper anterior			Lower anterior		
*(mm)*	**Malay (%)**	**Chinese (%)**	**Indian (%)**	**Malay (%)**	**Chinese (%)**	**Indian (%)**
** < -2**	15.4	3.7	9.1	1.9	5.6	4.5
**-2 to -1.5**	11.5	3.7	15.9	1.9	14.8	6.8
**-1.5 to -1**	13.5	7.4	6.8	0	11.1	6.8
**-1 to -0.5**	11.5	14.8	11.4	9.6	13	11.4
**-0.5 to 0**	15.4	18.5	13.6	19.2	7.4	13.6
**0 to 0.5**	15.4	7.4	11.4	17.3	24.1	18.2
**0.5 to 1**	9.6	9.3	9.1	17.3	13	9.1
**1 to 1.5**	3.8	11.1	9.1	17.3	7.4	18.2
**1.5 to 2**	0	3.7	2.3	3.8	0	2.3
** > 2**	3.8	20.4	11.4	11.5	3.7	9.1

**Table 6:** Percentage of subjects according to ethnicity requiring upper and lower overall correction

Correction	Upper overall			Lower overall		
*(mm)*	**Malay (%)**	**Chinese (%)**	**Indian (%)**	**Malay (%)**	**Chinese (%)**	**Indian (%)**
** < -2**	19.2	13	11.4	1.9	24.1	9.1
**-2 to -1.5**	15.4	5.6	4.5	3.8	5.6	11.4
**-1.5 to -1**	13.5	3.7	11.4	5.8	5.6	15.9
**-1 to -0.5**	11.5	11.1	15.9	9.6	9.3	9.1
**-0.5 to 0**	15.4	7.4	2.3	3.8	14.8	9.1
**0 to 0.5**	3.8	11.1	9.1	15.4	7.4	6.8
**0.5 to 1**	9.6	13	9.1	13.5	13	15.9
**1 to 1.5**	3.8	5.6	13.6	17.3	3.7	9.1
**1.5 to 2`**	1.9	1.9	9.1	9.6	5.6	2.3
** > 2**	5.8	27.8	13.6	19.2	11.1	11.4

Table 5 show the percentages of subjects in terms of the upper and lower corrections in millimetres which would be required to give the mean ratio for Bolton’s original sample. In these figures, a positive ( +) sign on the x axis indicates that the correction to be done is to increase the tooth structure—relative tooth size deficiency, whereas the negative (-) sign indicates that the required correction is to reduce the tooth structure—relative tooth excess.

For overall ratio as shown in Table 6, more subjects from Malay ethnic group presented with maxillary excess and mandibular deficiency with the equal percentage of 19.2%. In Chinese, maxillary deficiencies and mandibular excess are the main problems observed with 27.8% and 24.1% of subjects respectively requiring either maxillary tooth addition or mandibular tooth structure reduction. In Indian ethnic group, almost similar finding with Chinese, more subjects presented with maxillary deficiency (13.6%) followed by mandibular deficiency and maxillary excess in 11.4% of samples respectively. Small percentage of subjects presented with mandibular excess consisting of 9.1% which would require mandibular tooth structure reduction or enamel stripping to obtain occlusal fit.

## Discussion

### TSD and ethnicity

In this study, in reflection of Bolton’s standard, the results show that Malay subjects have a significantly greater prevalence TSD than the Chinese or the Indians. Because the type of malocclusion does not predispose a patient to a TSD problem, the differences between our results of Malay mean and Bolton’s cannot be explained by the orthodontic samples used. Bolton’s anterior and overall ideal ratios were significantly smaller than of the estimates in the current study of Malay subjects, except those for Chinese and Indian subjects. This explained the fact that Bolton’s original sample which has been composed primarily of Caucasian samples and implies that the Bolton ratio is only applicable to Malaysian Chinese and Indians it is not applicable to Malay population.

A few other similar studies agree with the findings of the present study, indicating specific tooth size standard for their populations; Spanish [16], Japanese [11], and Iranian-Azari [17] of both the anterior and overall ratios. Ta et al., [3] found that Bolton’s standard is only applicable to their Class I occlusion of Southern Chinese but not to those of Class II and Class III occlusion. Whilst Smith et al., [18] who studied was on Bolton’s applicability on three populations; Black, Hispanics and White, found that Bolton ratios apply to white females only; the ratios should not be indiscriminately applied to white males, blacks, or Hispanics. They also concluded that interarch tooth size relationships are population and gender specific. In this study, we found no significant difference for gender (Table 1**).**

### Frequencies of Bolton TSD

Proffit [19] quoted that prevalence of TSD in the general population as being 5%. Othman and Harradine [20] noted that the basis for this prevalence was not explained and maybe defined as the proportion of cases that will fall outside 2SD from Bolton’s mean ratios. Based on this definition, sample of this study consist of 20.7% of anterior ratio TSD and 4.7% prevalence of overall ratio TSD. It was found to vary among different findings of different populations.

There was higher incident of Malaysian ethnics’ subjects that fall beyond 2SD of Bolton anterior ratios as compared to overall ratio. Our findings are in agreement as that found by Crosby and Alexander [21] when comparing (3–3) anterior and (6–6) overall ratios, the authors found that in every malocclusion group, there is a greater percentage of patients with anterior mesial-distal TSD greater than 2SD from Bolton’s mean as compared with patients with overall discrepancies. The higher prevalence of anterior TSD seems to be quite agreeable to most authors [8, 21–24].

This is true whether we are looking at maxillary or mandibular tooth size excesses and results from the present study with findings from Crosby and Alexander [21]. This could be explained by the fact that anterior teeth, especially incisors, have a much greater incidence of tooth size deviations. The greatest variables in mesio-distal tooth width occur in the anterior region.

In the study, it remains questionable which deviations of the overall and anterior values proposed by Bolton affect the final treatment outcome. Peg-shaped lateral incisors are easily detected and express most often as TSD. Smith et al. [18] has similarly suggested that maxillary lateral incisors have previously been shown to be quite variable in size and are frequently the reason for a TSD between arches. And in this matter, aesthetic correction (crowns, veneers, etc.) is often the first choice of treatment. Hidden TSD from a generalized discrepancy in tooth width between upper and lower teeth is less detectible at first sight and may cause a less favorable treatment outcome. Because the present sample consisted of patients who were in the orthodontic waiting list with high orthodontic treatment need, the presence of a larger percentage of TSD than that in Bolton’s sample seem reasonable.

It is possible that the population of patients may be more diverse in the city of Kuala Lumpur with high migration rate from all over the country concentrated in the capital of Malaysia of which majority of the center’s orthodontic patients came from. Thus, the findings of this study may also represent the whole population in the country. It would seem logical that the percentage of patients that present with TSD may be somewhat dependent on the selection process or the characteristics of the population from which the subjects are drawn as observed in this study.

### Discrepancy in millimetres as a measure of clinical significance

Threshold of 2 standard deviations (SD) of Bolton’s mean ratios as clinically significance have been well accepted and documented [8, 20–23, 25]. The three ethnics displayed similar trend out of their sample which fell beyond the 2 SD (19.2% of Malay, 20.4% of Chinese, 22.8% of Indians) as shown in Table 4**.** This figures of clinically significant are in agreement as reported by other studies [19] but the incidence of overall ratio that fall beyond 2SD in current study of Malay and in Chinese sample are less than reported by this author which are between 5–14%. In this study, it was found that our sample displayed a lesser percentage of standard deviations of overall ratio in 1.9% in Malay and 3.7% in Chinese but a comparable amount as reported by [19] of 4.7% in Malaysian sample and 9.1% in Indian.

Endo et al. [25] mentioned the importance of TSD expressed in terms of both percentage and amount of millimetres required for correction. The authors quoted ‘ratios outside 2SD and TSD requiring more than 2 mm of maxillary and/or mandibular corrections are recommendable as the appropriate thresholds for clinical significance’. Othman and Harradine [20] had raised the issue of the fundamental of an absolute size of discrepancy thought to be incompatible with an acceptable occlusal fit.

Proffit suggested that 1.5 mm as a cut-off point to be clinically significant [26]. This has been accepted by Bernabe et al., [8] in his study as their limit of acceptable discrepancy. A TSD of less than 1.5 mm is rarely significant, but larger discrepancies create treatment problems and must be included in the orthodontic problem list at treatment planning stage [26]. In the current study, subjects requiring more than 2.0 mm corrections of either arches were considered of clinically significant as recommended by recent studies [19, 25] as a threshold for clinically significant TSD.

Bernabe˘ and coworker [8] stated that the frequency of subjects with clinically significant TSD differs based on which arch is considered “normal”. If mandibular tooth-width is defined as normal, a tooth discrepancy would be described as a maxillary tooth-width deficiency or excess. From Table 4 illustrating percentage of subjects with clinically significant tooth-width discrepancies larger than 2.0 mm of Malaysian samples, it was found that in anterior as well as overall ratio, more subjects possessed a maxillary excess than other problems.

However, from Tables 5 and 6, illustrating the clinically significant tooth width discrepancies according to ethnic group, it was found that Malay subjects present with more problem located in the maxillary arch due to maxillary excess in both anterior (26.9%) and overall (34.6%) ratios complemented by mandibular tooth deficiency in both arches (14.3% and 28.8% respectively).

As opposed to findings in Malay subjects, Chinese subjects presented with more problems in the mandibular arch, due to mandibular excess (20.4%, 29.7%) and maxillary deficiency (24.1%, 29.7%) in both anterior and overall ratios respectively (Table 4). Indian subjects on the other hand presented with equally fair distributions of all problem area with main problem of anterior ratio located 25% in the maxilla due to excess and locating overall ratio problem due to maxillary deficiency in 22.7% of subjects. McLaughlin et al. [27] found that there are more common to find an excess of tooth substance in the lower arch as found in the Chinese sample in this study. The authors mentioned that it is usual to reduce tooth mass in the lower incisors by inter-proximal enamel reduction and/or by addition of tooth mass with restorative materials in the opposing arch of upper incisors, commonly the laterals.

In the present study, high incidence of mandibular excess and maxillary deficiency in our Chinese samples could be contributed by high prevalence of Class III malocclusion found among Chinese. This is supported by Woon et al. [28] that there was a high percentage of edge to edge incisor relationship in Chinese (54%), followed by Malay (50%) whilst the Indian had 38%.

In this study we are comparing the malocclusion using BSI classifications which only relate the relationships between the upper incisors and lower incisors’ edges and found no correlation of TSD with malocclusion class **(**Table 2**)**. This finding is in agreement with Laino et al. [29]. However, our study is in dispute with a study by Mulimani et al., [30] which found a significant correlation between malocclusion classes. However, Alshahrani found no significant difference in both ratios among the various malocclusion classes in Southern Saudi subjects [31].

Malay samples displayed more maxillary tooth excess as presented in Table 4. It was reported [28] that Malay presented with the highest percentage of increased overjet problem (16.11%) as compared to Chinese (12.75%) and Indians (9.52%). The greater prevalence of TSD among the Malay subjects are due to genetic and strong inheritance pattern. The origin of Malay that can come from diverse origin and ancestry can contribute to this disproportion of tooth size even though, subjects with mixed marriage was not selected in this study.

In clinical practice, clinicians often note the discrepancy of tooth size and skeletal size but seldom pay attention to TSD between maxillary and mandibular teeth. From this study, it might be reasonable for orthodontists to denote interproximal stripping or tooth extraction in the mandibular dentition for mandibular tooth size excess which is common in Class III malocclusion and in the maxillary dentition for maxillary tooth size excess, a common presentation of Class II malocclusion.

These results suggested that the Bolton analysis is important and should be considered when diagnosing, planning, and predicting prognosis in clinical orthodontics. With relatively high tooth excess with the opposing arch, it is another factor to consider for extractions decision. Bolton himself had stated the effects of extractions that can reduce the existing high anterior or overall ratios and this are supported by Saatci and Yukay [32].

The limitation of this study is that the measurement of tooth size discrepancies was measured using the digital calipers with HATS software on study model instead of a digitized model using the digital scanner. However, Correia et al. [33] found no statistically significant differences between manual and digital methods for measuring tooth size discrepancies, except for values found by the linear digital method which revealed a slight, non-significant statistical difference.

This study sample does not represent the ratio of the ethnicities as the study did not conclude Malaysian norm. The values derived from this study were to represent the TSD using Bolton’s analysis for the major ethnicities in Malaysia. A cross sectional study design was adopted when comparison of each ethnicity of the Orthodontic clinic in Kuala Lumpur was made. Bolton’s study [1] consisted of 55 study casts to represent the American population of 331 million compared to our sample (150 subjects) which are considered plentiful to represents 33 million of the Malaysian population.

## Conclusion

There was a significant difference found between the TSD of the three major ethnicities in Malaysia. The Bolton standards can be applied to Malaysian Chinese and Indians but not to Malays orthodontic populations for both anterior and overall ratios. Subsequently, a specific standard should be used for the Malays orthodontic population which is found in this study. It was found that more Malay subjects presented with maxillary excess contrary to Chinese and Indians who presented more maxillary deficiency for the anterior and overall ratio.

## Data Availability

All data generated or analysed during this study are included in this published article [and its supplementary information files]. The datasets used and/or analysed during the current study are available from the corresponding author on reasonable request.

## Abbreviations

TSD: Tooth size discrepancy
ANOVA: Analyses of variance
HATS: Hamilton Arch Tooth System
SPSS: Statistical Package for Social Sciences
SD: Standard Deviations
